# Research partnerships between high and low-income countries: are international partnerships always a good thing?

**DOI:** 10.1186/s12910-015-0030-z

**Published:** 2015-05-28

**Authors:** John D Chetwood, Nimzing G Ladep, Simon D Taylor-Robinson

**Affiliations:** Department of Hepatology, 10th Floor QEQM Building,St Mary’s Hospital, London, W2 1NY UK

**Keywords:** International Health, Development, Nigeria, Collaborative research, Global Partnership

## Abstract

**Background:**

International partnerships in research are receiving ever greater attention, given that technology has diminished the restriction of geographical barriers with the effects of globalisation becoming more evident, and populations increasingly more mobile.

**Discussion:**

In this article, we examine the merits and risks of such collaboration even when strict universal ethical guidelines are maintained. There has been widespread examples of outcomes beneficial and detrimental for both high and low –income countries which are often initially unintended.

**Summary:**

The authors feel that extreme care and forethought should be exercised by all involved parties, despite the fact that many implications from such international work can be extremely hard to predict. However ultimately the benefits gained by enhancing medical research and philanthropy are too extensive to be ignored.

## Background

The interest and ability to develop international partnerships in the pursuit of research goals is greater than ever, especially with advances in technology and an increased cultural awareness of common issues that face everyone around the world.

It is now nearly universally accepted that with increasingly mobile populations, health issues are less parochial and are of global concern, given that cultural, social health factors and diseases transcend borders. Communicable diseases are not restricted by geography or borders with ever growing migration as the current Ebola outbreak illustrates; the NHS continues to look for low cost innovation, especially with recent economic stagnation; and globalisation means the UK is increasingly less isolated in health and economic terms. Thus, ‘Global Health’ has burgeoned from its perception as a niche subject to core knowledge, as reinforced by ‘The Gold Guide’ [1], ‘Broadening Your Horizons’ produced by the BMA [2], and ‘Tomorrow’s Doctors’ produced by the GMC [3], and the increasing presence on undergraduate and postgraduate medical curricula.

There is also humanitarian need for international research, as medical initiatives in low-income countries are continually underfunded and underrepresented in clinical medicine and research programmes, and the greatest potential to improve life expectancy and quality lies there – for example, with neglected vaccination efforts for viral hepatitis B. However, there are certain risks and considerations to international collaboration and the influence from wealthy institutions. Research findings from a higher-income country may be costly or hard to implement in a lower-income country, may not be culturally appropriate, and often these focus on non-communicable diseases, prevalent in northern countries. Therefore, there can be a tendency to neglect the specific local needs of lower-income countries.

In 2002, the Nuffield Council on Bioethics published its report “The Ethics of Research Related to Health Care in Developing Countries” [4]. This departs from the internationally accepted Declaration of Helsinki in that it argues it may be morally acceptable to establish paradigms more applicable to the location of study albeit the ‘best intervention currently available as part of the health system’, rather than developed world gold-standards.

However, such local standards are often aspirational, rather than grounded in practicality, and open to interpretation given a lack of national standards, protocols and algorithms, particularly where the realities of healthcare depart from ideal guidelines.

In this article we examine the advantages and pitfalls of such international work.

## Discussion

### Refocusing research resources

In moral terms, international collaboration should move research goals away from pure market forces and towards humanitarian aims. The greatest need for increasing quality and length of life lies in low-income countries and cooperation may result in a marriage of a high-income world’s research capacity with access to a low-income country’s population and health needs, for mutual benefit. This problem of disproportionate spending on health research, which neglects the poorest populations, came into notoriety with the “10/90 gap” publications from the Global Forum for Health Research (GFHR) [5] and has persisted [6] despite the increasing role of such philanthropic foundations as the Wellcome Trust [7] and the Bill and Melinda Gates Foundation [8].

First, this allows efforts to focus on debilitating diseases that can have great improvements in health factors from small investment rather than those which simply provide good financial returns, as exemplified by such private-public collaborations as the Medicines for Malaria Venture (MMV), and organisations such as the Schistosomiasis Control Initiative, which is consistently rated as one of the most cost-effective charities in existence [9-13]. Second, this provides commercial viability to research that might not otherwise be possible, where afflicted populations are generally impoverished and underserved by pharmaceutical interest, such as the aptly named ‘neglected tropical diseases’ [14,15]. Third, with international funding, expertise and equipment, there is an increased scope in what the research itself may achieve, for example with genetics or metabolomics which require expensive equipment and specialist technicians, but where diseases are more prevalent in lower-income countries, concentration on these populations may lead to quicker recruitment of patients. Last, international research may better control for genetic, social and cultural bias present, than if examining just one population. Information from larger areas and a larger span of populations ensures a greater accuracy to the work itself. Multicentre research validating metabolomic and proteomic biomarkers in liver disease, across diverse populations is an example of just such a success story [16-18].

Nonetheless, such distortion, even if intended to be philanthropic, can skew research outcomes to a high-income country’s agenda [19]. Higher-income countries institutions may lack insight into a lower-income partner country’s infrastructure, method of practice and make any gains inefficient, or even impossible to be implemented [4,20]. There also concerns that cultural differences can lead to unintended ethical considerations such as whether true informed consent is being collected [4,21], or what intervention continues after a study ends [4]^,^ or whether research efforts could be detracting from other potential opportunities such as public health provision.

Examples of such healthcare inequalities, however well meaning, are seen with international HIV-AIDS work, owing to its relative funding, compared to other more under-resourced diseases. WHO and World Bank resources have been directed to provide heavily subsidised ($20 per annum) or free HIV treatment, but many of these drugs, such as tenofovir and lamivudine, are also active against hepatitis B, a condition that does not receive any subsidised funding. For example, in West Africa, where deaths from the complications of hepatitis B, such as hepatocellular carcinoma are very common, patients with hepatitis B monoinfection must pay for their own antiviral drugs. This is prohibitively expensive for the majority of people, and often not regularly feasible especially for more expensive, newer agents on patent, such as tenofovir, which can cost upwards of $8000 per annum. However, if an individual contracts HIV, then their drugs, including antivirals, such as tenofovir, are externally paid for by the Global Fund and become free at the point of care [22,23]. In other words, such inequalities in funding have a market distortion in poorer African populations, where contracting an HIV superinfection to viral hepatitis may improve their prognosis, given that HIV positivity allows access to definitive treatment for viral hepatitis too.

Similarly international partnership between the North and the South may also cause the transfer of resources away from governmental and market forces that encourage delivering healthcare, and into academically-driven research-directed goals that do not directly and immediately benefit the population of low-income nations – especially where national research aims are not clearly established or adhered to. This is particularly relevant when results are published in peer-reviewed journals, but the countries in which it may make the most impact may not have access to these journals [24], or speak the same language - despite the rise in open access journals [25], free online access networks [24] and international journal partnerships (such as JAMA and the Lancet which have created links with African journals in order to boost African research availability).

Ultimately, all research teams active in the international health arena should provide research questions that are either in line with lower-income countries’ national priorities, or they should be in a position to justify to external bodies the reasons why agendas do not comply with criteria set out in the Nuffield report [4]. Conflict with different aims for research work may be avoided with transparency and good communication.

### Infrastructure and capacity building

Investment from external research bodies, including industrial partners, may allow the development of local capacity. Foreign investment can also stimulate demand for goods and services and therefore economic growth, even those not directly associated with the research work itself (for example local courier companies for sample transport). This is in line with Keynesian view of macroeconomics in the assertion that demand will stimulate greater production to enter the market and therefore a growth in the net size of the economy. A greater wealth of the economy and therefore wealth per capita, allows a greater expenditure on goods and services that can benefit the individual (including in health), as well as allows greater taxation revenue for public provision, with greater marginal benefits seen at the poorest ends of the spectrum.

Material investment is important in increasing research structure and research potential (especially as countries with higher GNP per capita generally spend a larger percentage and absolute amount on medical research and development [26,27]), but also critically, international research causes the dissemination of knowledge, incentivising up-to-date information and standardisation of clinical practice. This effect is pronounced with international healthcare studies with work opportunities afforded as a result of the relationships developed. This can be beneficial, both for low-income country staff when they return with expertise in clinical and research training, but also for high-income country staff, given that access to populations in low-income countries with increased disease prevalence allows research targets to be reached more quickly. The Tropical Health and Education Trust (THET) is one such organisation that creates a framework for ‘health partnerships’, which are ostensibly mutually beneficial. Such collaborations not only nurture the dissemination of knowledge, but also can build on multicentre research aims and opportunities.

On the other hand, when international placements become feasible in research establishments in the North, there is a risk of causing a ‘brain drain’, where skilled and talented labour is permanently exported, depriving the domestic healthcare system in lower-income countries. This has contributed to situations, such as in Malawi where the doctor to population ratio in 2008 was just 1.9 doctors to 100 000 population [28], compared to the UK’s 2400 [29] (although this issue is undoubtedly multifactorial including training opportunities, provision of public resources, poor equipment in rural areas, low pay, bureaucratic red tape and political interference [30-32]). However the brain drain issue is contentious, despite its clear rationale as an argument, as staff may be viewed, as commodities, neither taking their wishes for self-determination into account, nor their quality of life.

Long-term placements in developed world institutions also run the risk of over familiarizing healthcare professionals with alien healthcare systems that provide inappropriate experience. Many migrant workers, who come to low-income countries for periods of research or clinical training, have skill sets that are appropriate for practice in their own countries, but all too often individuals find themselves ineffective and inexperienced in inappropriate surroundings, and such experience in the North may not improve their professional capacity if they return home, given that disease prevalence and treatment guidelines may differ. This may limit employment opportunities if domestic employers suspect returnees of being unfamiliar or with outdated knowledge of accepted methods of local clinical or research practice.

Medical expertise may also be retained in Europe, North America or Japan. For example, if biomedical samples are exported for analysis using expensive equipment in high-income countries, there may be little incentive to train local people in analytical techniques. In an effort to develop division of labour, personnel from lower-income countries may be practicing uncomplicated tasks that hinder their opportunity to engage in novel research techniques available in their native institutions. As Costello et al. [33] argue, ‘postal’ (sending biomedical samples) or ‘parachute’ research (sending researchers on short-term placements) may limit low-income countries involvement in the research and provide an opportunity cost of the collaboration, if there is not a concomitant sharing of the analysis.

In addition, research may have political, economic or societal impacts beyond what was planned, especially where cultural and social beliefs are ignored and where healthcare aims are hijacked to high-income countries’ agendas. Unfortunately, high-income institutions often take advantage of low-income countries in setting agendas and may give the latter little or no credit in leading international health partnerships. Furthermore, if there is commercial value in the intellectual property then low-income country institutions can be exploited financially as well as intellectually [34].

Ultimately, it is often unlikely for many low-income countries that the funding or infrastructure exists to develop capacity without timely economic development and long-term health structure investment. Although risks must be considered, it may provide unique effective methods to improve global quality of life.

### Producing high class research

As explained above, true partnership in international health allows access to more diverse patient groups, with different ethnicity and health behaviours. Larger patient groups may enable enhanced patient recruitment, especially with rare diseases such as with Burkitts Lymphoma research in the 1950-60s [35], and can control for a larger variety of local factors with the potential to create higher powered research results.

Reputable high-ranking institutes may also lend credibility to research from high-income countries that would otherwise be ignored as irrelevant or viewed with suspicion in peer-reviewed journals when taking into account the material’s source [36] (although true peer-review should predominantly focus on the quality of the work itself), and bring important research into a more commonly spoken language or higher impact-factor journals in wider circulation and therefore to a wider audience [37].

Although there is often a financial cost to international partnership with two-way visits and inspections, material and sample transport between sites, and adherence to ethical and/or clinical guidelines, relative costs, especially for labour are often lower. The cost of international collaboration depends on the research involved. Financial aspects become more problematic either when relationships crumble, sometimes leading to a significant loss of time and money, or when exchange rates fluctuate which can lead to unforeseen instability in budgetary planning.

Research across borders may also call for adherence to different guidelines, with multiple ethics committee approvals that increases red tape and make collaboration difficult. However, this is really only a small barrier to collaboration, rather than an argument against it.

There is often a perception that medical research in lower-income countries takes place in areas with less rigorous safety and ethical safeguarding to the detriment of patient, and where errors are permitted to occur more frequently. Although such a statement is so generalised as to sometimes be true, frequently perceptions that low-income countries cannot carry out high-class research may lead to more stringent guidelines and safeguards. This, in turn, can hinder an institution’s ability to carry out research. Furthermore, this is mostly avoided when many institutions carry out independent ethical review in the partnering high-income country.

## Summary

International research can certainly be problematic, and will introduce foreseeable and unforeseeable difficulties. Such efforts tend to fall on a spectrum - few people would condone such work as AZT testing in Zimbabwe in the 1990s [38] or the Turkegee experiments in the USA [39], but without international collaboration neither would we have the success stories of small pox or polio vaccination.

With rigorous planning, good ongoing communication and transparency, adherence to internationally-accepted ethical standards, and clear intended outcomes, common problems can often be avoided. The benefits gained by enhancing medical research and philanthropy are too extensive to be ignored, but do require extreme care and forethought, despite the fact that many implications from such international work can be extremely hard to predict.
